# Identification and Surgical Management of Upper Arm and Forearm Compartment Syndrome

**DOI:** 10.7759/cureus.5862

**Published:** 2019-10-08

**Authors:** Adel Hanandeh, Vishnu R Mani, Paul Bauer, Alexius Ramcharan, Brian Donaldson

**Affiliations:** 1 General Surgery, Columbia University College of Physicians and Surgeons at Harlem Hospital Center, New York, USA; 2 Surgery, Columbia University College of Physicians and Surgeons at Harlem Hospital Center, New York, USA; 3 Surgery, Harlem Hospital Center, New York, USA

**Keywords:** upper arm compartment syndrome, fasciotomy, forearm compartment syndrome, condylar fracture, pediatric supracondylar humerus fracture

## Abstract

Extremity muscles are grouped and divided by strong fascial membranes into compartments. Multiple pathological processes can result in an increase in the pressure within a muscle compartment. An increase in the compartment pressure beyond the adequate perfusion pressure has the potential to cause extremity compartment syndrome. There are multiple sites where compartment syndrome can occur. In this article, an arm and forearm compartment syndrome ensued secondary to a minor crushing injury that lead to supracondylar and medial epicondylar non-displaced fractures. A pure motor radial and ulnar nerve deficits noted on presentation, worsened with progression of the compartment syndrome. Ultimately, a surgical fasciotomy was carried out to release all compartments of the right upper arm and forearm.

## Introduction

In 1872, the first description of compartment syndrome was published by Richard von Volkmann. His publication described an irreversible contracture of muscles due to ischemic process resulting in the first documented nerve injury and contracture from compartment syndrome. “The tight bandage, aided by the tight skin and fascia, that does the mischief” a famous statement by J.B. Murphy (1914) who was the first to suggest that fasciotomy done early might prevent contractures from developing [1-5].

Compartment syndrome is a well-known surgical emergency with high morbidities resulting in potentially long-term debilitation [1-2]. While compartment syndromes are not unusual, occurrence in certain parts of the body, such as the arm and or buttock, can be extremely rare resulting in delayed diagnosis or mismanagement. Hence, an immediate recognition can lead to early management and much better outcome [3].

Multiple compartment syndrome causes have been reported in the literature including long bone fractures, acute extremity ischemia with reperfusion, burn injury, crush injury, soft tissue infection, myositis/myonecrosis/rhabdomyolysis, systemic inflammatory response syndrome (SIRS), massive fluid resuscitation, snake bite and prolonged immobilization [1-3].

It is important to note that most symptoms including nerve conduction disturbances emerge when the delta pressure is less than 30 (the difference between the compartment and diastolic pressures) or when the compartment pressure becomes greater than 30 mmHg [5-6].

An upper arm compartment is a rare type of compartment syndrome. There have been multiple causes of upper arm compartment syndrome described in the literature including traumatic and non-traumatic causes [3,7]. Traumatic causes included bone fractures, crushing injuries, and vascular injuries. Non-traumatic causes included pneumatic tourniquet at the upper arm, prolonged extremity immobilization, soft tissue infection, myositis and other processes that can lead to muscle necrosis and after venipunctures [3-4,8].

Upper arm and forearm compartment syndrome can lead to significant sequelae including severe limb ischemia requiring amputation, contracture development, severe nerve injuries including permanent motor and or sensory deficits [1-4].

## Case presentation

A 25-year-old male construction worker with no past medical history presented to emergency room (ER) 30 minutes after sustaining a right elbow crushing injury by a compactor at work. The patient did not report any significant past medical or surgical history, and he also denied any allergies or taking any medications.

Review of systems (ROS): significant for right arm and forearm swelling, pain, three open wounds on the medial/lateral aspect of the arm with minimal bleeding, and limited right-hand range of motion. Vitals signs: BP 121/78, HR 75, RR 18, Spo2 100% on RA, temperature 98.7F. Constitutional: Alert and oriented x3. Musculoskeletal: marked swelling of right forearm and elbow area, 2.5 x 1 cm gaping wound on the distal right upper arm, 1 x 1 cm laceration with minimal bleeding on the proximal right arm, 1 cm linear laceration on right proximal forearm, + eccymosis, 2+ pulses. Neuro: radial and ulnar neuropathy - unable to extend the right wrist, extend, flex, abduct, or adduct digits of the right hand. Labs included a normal complete blood count (CBC), prothrombin time/partial thromboplastin time (PT/PTT), basic metallic panel (BMP), and significant for an elevated creatine kinase (CK) to 582.

**Figure 1:**
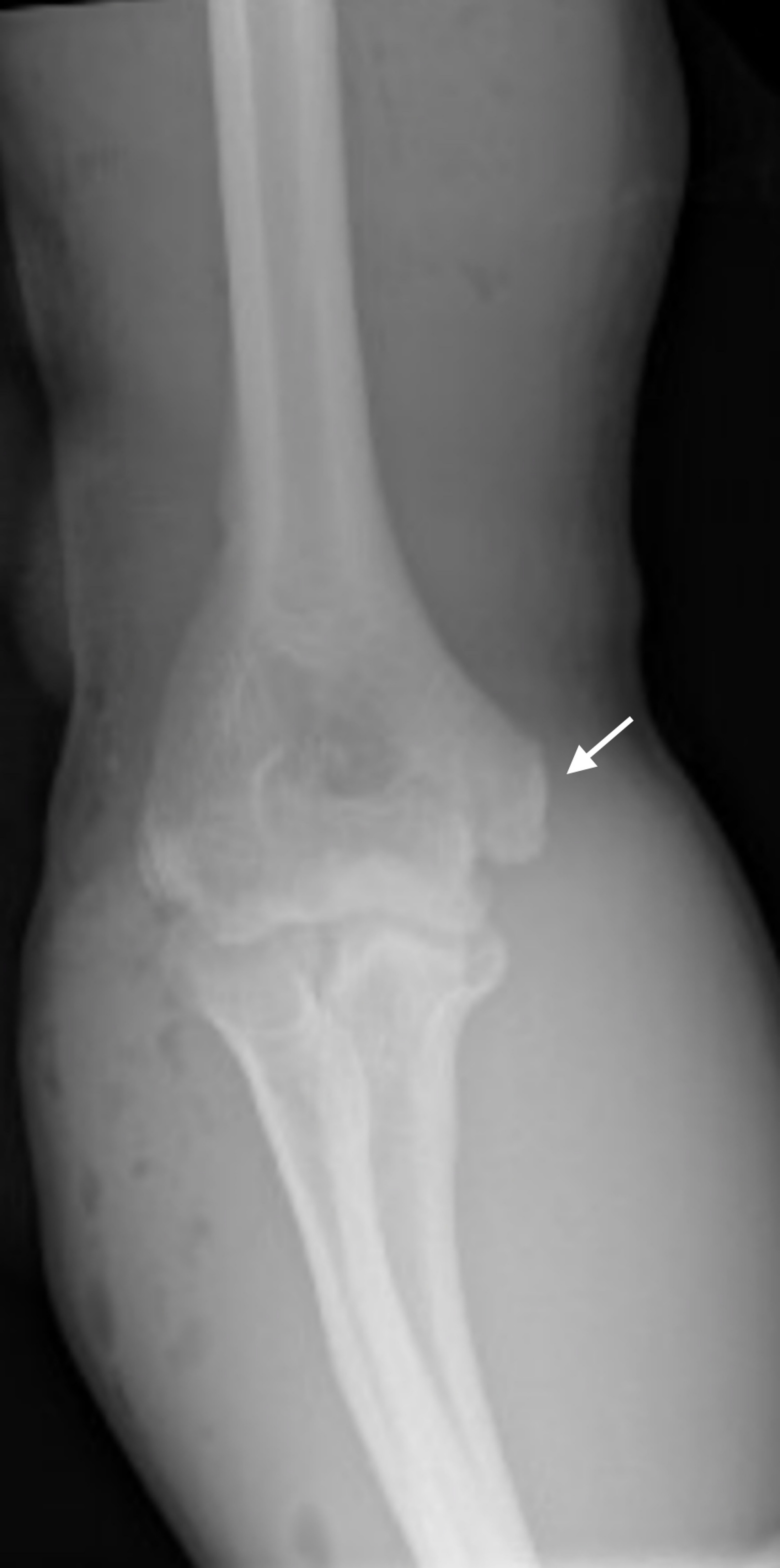
Cortical fracture of the distal humerus with adjacent soft tissue swelling and subcutaneous emphysema

Imaging included an anteroposterior (AP) and lateral X-ray of the right humerus illustrated cortical fracture of the distal humerus with adjacent soft tissue swelling and subcutaneous emphysema and possible avulsion of medial epicondyle of distal humerus (Figure 1).

Computed tomography angiography (CTA) was also done which indicated no gross evidence of acute vascular injury, no evidence of a pseudoaneurysm, or evidence of extravasation of the administered intravenous contrast. However, there was moderate nonspecific decrease caliber of the dorsal interosseous arterial branch, possibly related to vasospasm, as well as a cortical fracture of the distal humerus, and avulsion fracture of the medial epicondyle.

**Figure 2:**
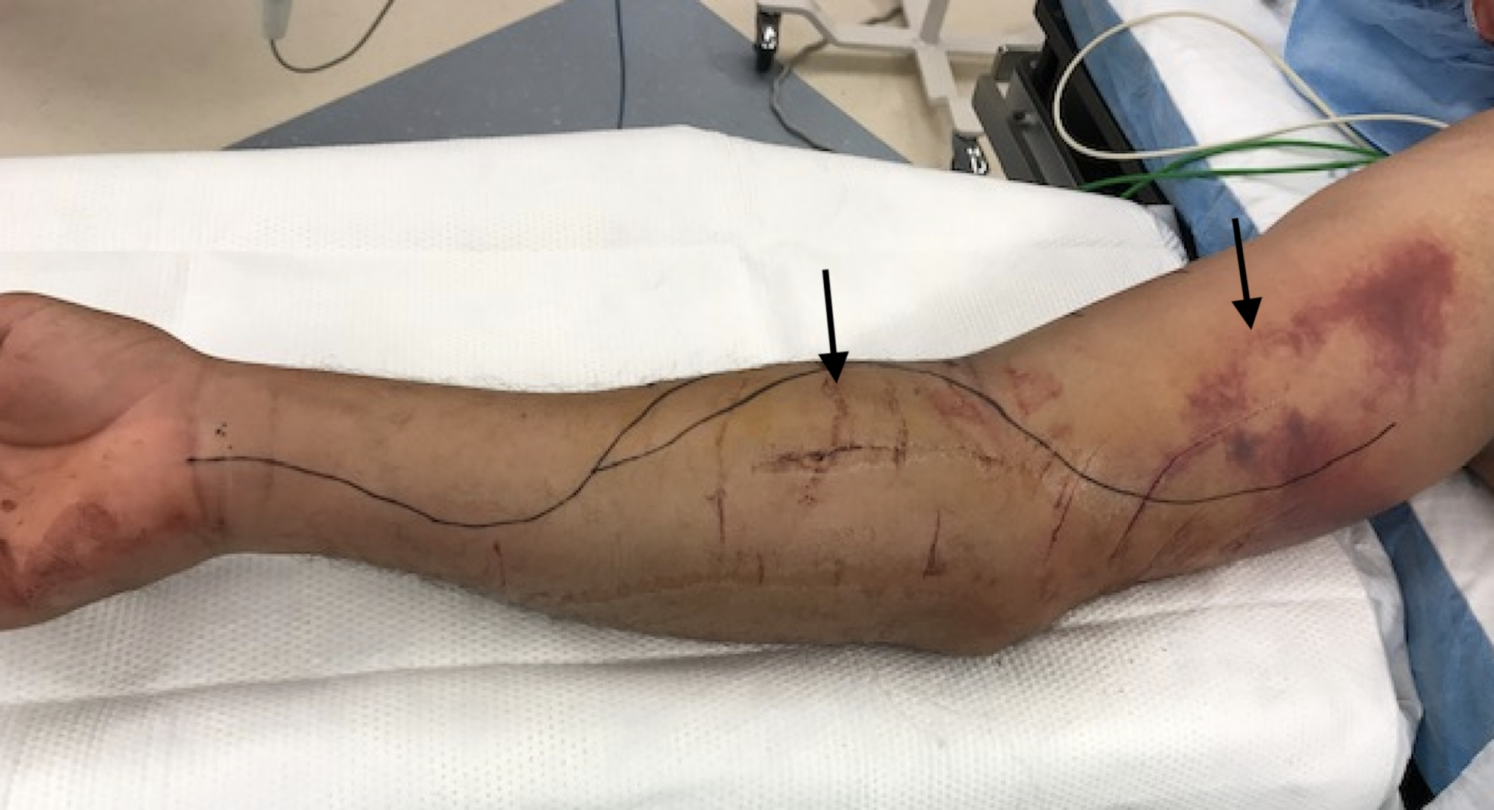
Right arm and forearm severe swelling

The patient was admitted for observation and pain control. Two days later, he was found to have severe pain in the right arm and forearm, severe swelling, and progression of the right extremity motor deficits (Figure 2). The patient also endorsed new onset of numbness, tingling and pain out of proportion to elbow flexion, or extension. On physical exam radial pulses were intact bilateral (b/l). Labs were significant for normal worsening elevation of CK to 1838.

**Figure 3:**
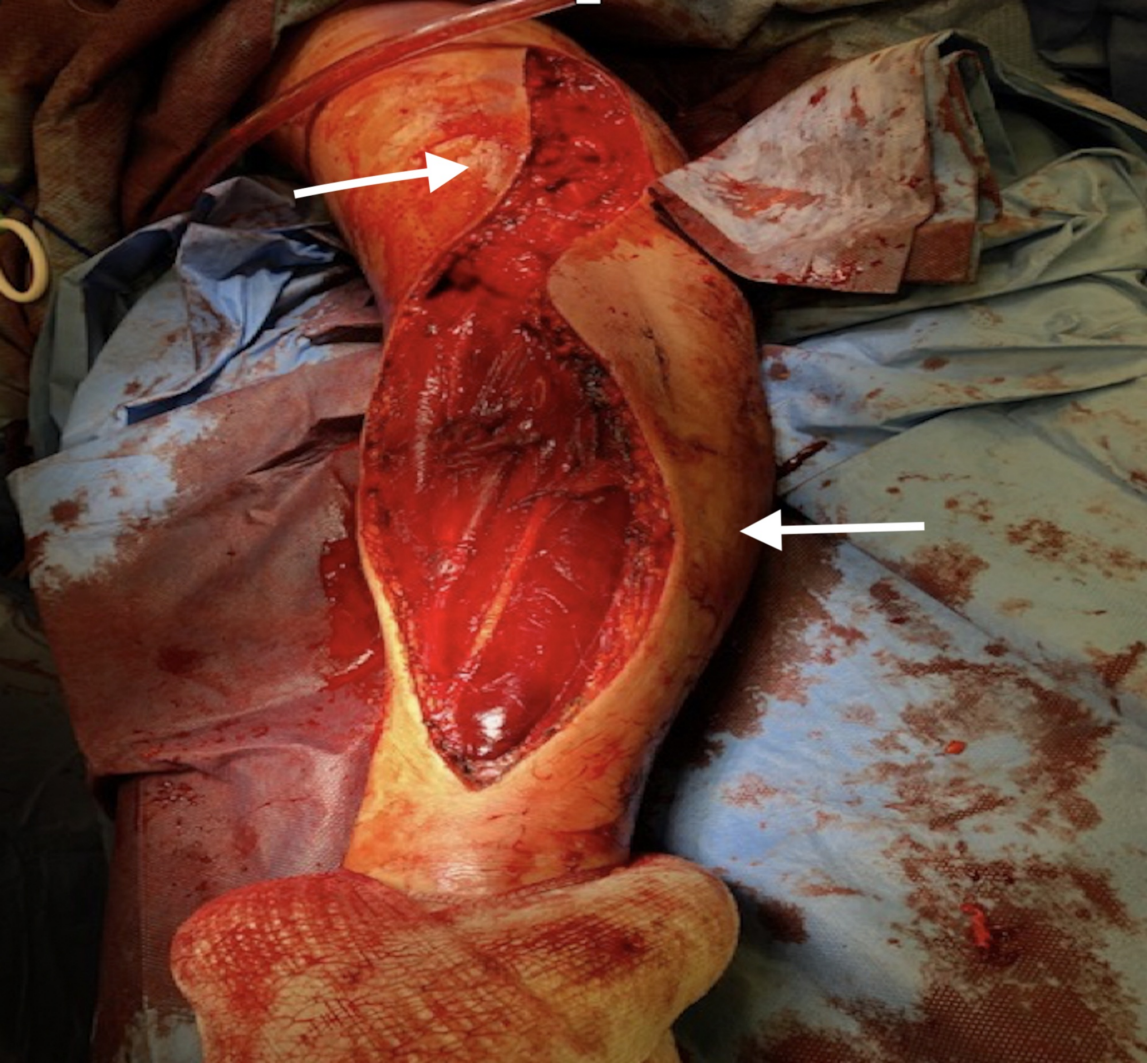
A lazy S-shaped incision was carried along the ventral aspect of the forearm without extension to hand, hence the flexor retinaculum ligament was not divided.

The patient was taken urgently to the operating room for a fasciotomy of right upper arm and forearm. A limited lazy S shaped fasciotomy was performed along the volar aspect of the forearm without dividing the flexor reticulum or right hand. An incision was made 1 cm proximal to the medial condyle and curved medially, reaching the midline at the junction of the middle and distal third of the forearm (Figure 3). Fasciotomy released the fascia of the superficial layer, the deep layer that contains the pronator quadratus, and the deep flexor compartment. The dorsal compartment and the mobile extensor wad were released without the need for a dorsal incision.

**Figure 4:**
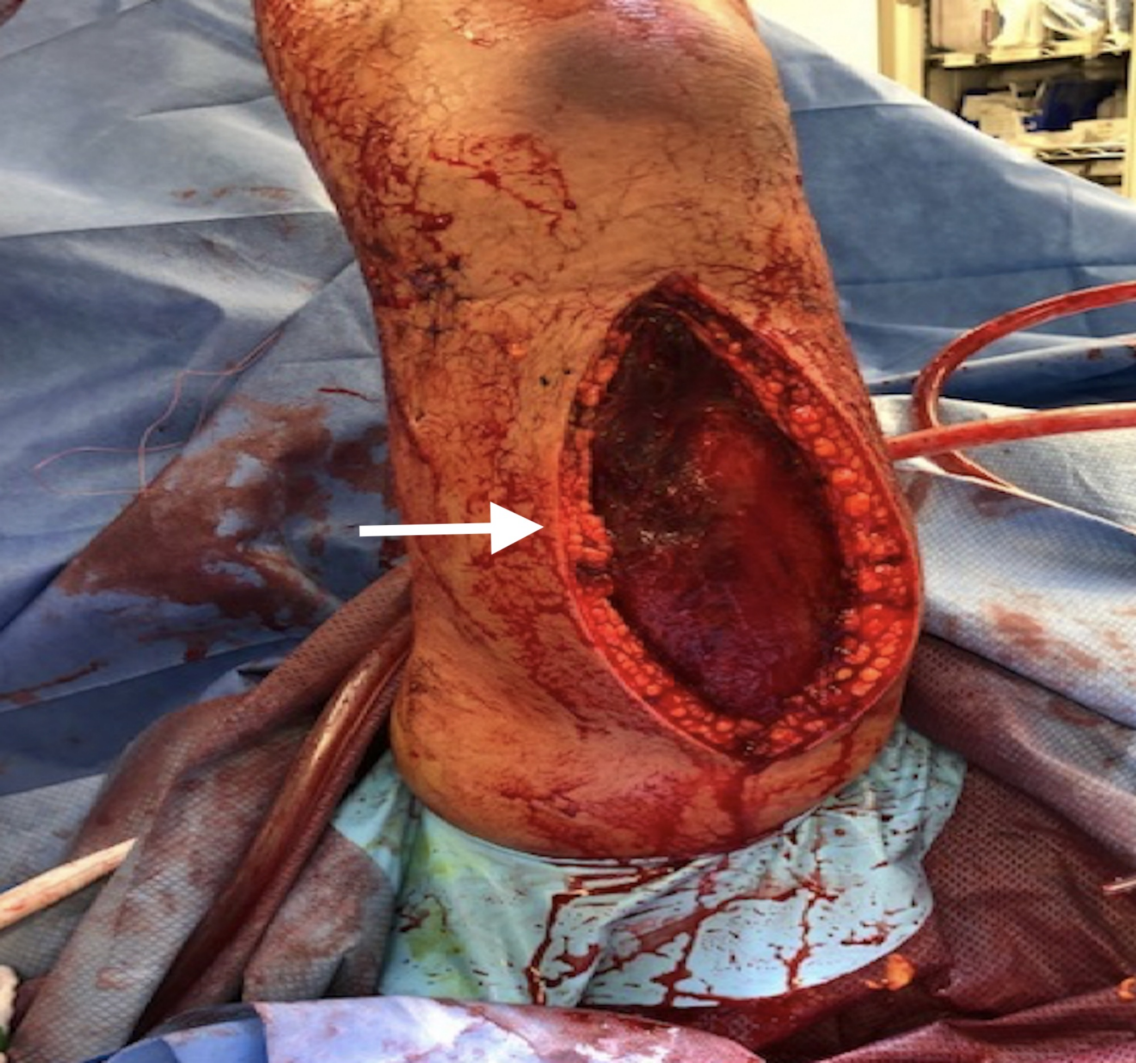
A 15-cm posterior vertical incision was carried along the posterior midline, allowing for evacuating blood collection in the posterior compartment.

The lazy S-shaped incision was extended superiorly into the upper right arm releasing the anterior compartment (Figure 3). A vertical dorsal incision was performed along the upper right arm allowing the release of the upper arm dorsal compartment (Figure 4). Upon surgical exploration of the right arm anterior and posterior compartments, a deep sub-fascial hematoma as well as right forearm deep volar and dorsal compartments subfascial hematomas were noted.

Postoperatively, on exam his right arm and forearm appeared soft with intact sensation and pulses. The patient continued to endorse pure motor deficit in the right hand with inability to extend his wrist extend the digits fully. He was also unable to abduct or adduct digits in the right hand. Neurology service was consulted, and the patient was found to have neuropraxia in the radial and ulnar nerves.

## Discussion

Compartment syndrome occurs most commonly secondary to a high-energy limb injury. Yet, trivial injuries can also lead to a compartment. Crushing injuries are among the most common causes of compartment syndrome. Young men appear to have the highest incidence which could be due to their larger muscle mass within fascial compartments [7,8].

An upper arm compartment is certainly among the rarest sites for a compartment syndrome. Upper extremity compartment syndrome occurs highest in patient with crushing injuries and humerus bone fractures [3,7].

All types of compartment syndromes share a similar pathophysiology which occurs when cellular anoxia is achieved. Due to the limited distensibility of the fascia that encloses the muscles any fluid volume expansion or expansion restrain within a muscle compartment can lead to increasing the internal pressure and ultimately compartment syndrome [1-3,5]. Thus, compartment syndrome occurs when the pressure within a closed osteo-fascial muscle compartment rises above a critical level. A critical pressure is the tissue pressure at which the capillary vessels collapse leading to low-pressure blood flow through the capillaries and ultimately restrict venous drainage [5-6]. Generally, the normal tissue pressure is between 0-10 mmHg. A vicious cycle ensues by restricting local tissue perfusion by reducing the arteriovenous pressure gradient (reduced arterial pressure, increased venous pressure) and, if prolonged, will result in cellular anoxia, leading to muscle and nerve damage [6].

A compartment pressure capable of compromising perfusion develops when it rises to within 10 to 30 mmHg of diastolic pressure; or when the compartment pressure is above 30 mmHg in which muscle oxygenation decreases as tissue pressure approaches mean arterial pressure [5].

As the compartment pressure continues to rise beyond this point, nerve conduction ceases and motor paralysis will occur [1-4]. Further progression of ischemia results in tissue necrosis including myocytolysis. The degree of muscle damage depends on the duration of extremity ischemia and the metabolic rate of the tissue, but generally irreversible damage ensues after four to eight hours [5]. Ultimately, long-term ischemia may lead to liquefying necrosis of the muscles within the compartment [6-8].

Compartment syndrome can be classified into acute or chronic based on their clinical manifestation. In chronic compartment syndrome, patients usually exhibit recurrent, transient increases in compartment pressures during exercise with transient neurologic symptoms and pain, which resolve with rest [9-12].

Acute compartment syndrome is usually further classified as impeding or established based on clinical features supported by measurement of compartment pressures. In impending compartment syndrome, tissue pressure increases and tissue perfusion is reduced, but is not sufficient to cause muscle or nerve damage. Established compartment syndrome occurs when the pressure in the compartment rises sufficiently to cause tissue ischemia generally greater than 25-30 mmHg. When pathologic tissue pressure elevation presents for less than four hours, acute compartment syndrome is defined as an early stage, and more than four hours is considered a late stage [1-5].

Compartment syndrome can develop as a result of any condition that increases the volume of the compartment without an increase in the diameter of the unyielding myofascial envelope [6]. Multiple causes have been reported including long bone fracture, acute extremity ischemia with reperfusion, burn injury, crush injury, soft tissue infection, non-traumatic myositis/myonecrosis/rhabdomyolysis, systemic inflammatory response syndrome (SIRS)/massive fluid resuscitation, snake bite and prolonged immobilization [1-5,8-12].

The diagnosis of a compartment syndrome is usually based on two main factors: a high index of suspicion and understanding of the variable clinical presentation. The earliest clinical symptom is pain out of proportion and pain with passive stretching of the muscles. Other symptoms, such as paresthesia, pallor, paralysis, poikilothermia and lastly pulse-lessness may occur as the pressure and duration progresses. Usually a sequence of diminished light touch followed by hypoesthesia, and finally progressive motor weakness are observed [6-8].

Compartment pressure is usually measured when the diagnosis is unclear hence pressure measurement can prevent unnecessary fasciotomy. But when the clinical presentation is obvious, there is usually no benefit from measuring pressures and immediate fasciotomy can be undertaken. There are multiple techniques to measure a compartment pressure including handheld manometer (stryker), mercury manometer, large-bore needle and connecting tubing (after Whitesides in 1975), an electronic strain gauge used for physiologic monitoring in ICU, or the wick or slit catheter technique [11-12]. Image studies including ultrasound, CT, and MRI are usually omitted in order to prevent any delay in treatment. The gold standard for treatment for an acute compartment syndrome is an emergent fasciotomy [1-4].

In this article the upper arm and forearm compartments are the main focus and understanding the anatomy of those compartment will lead to a successful diagnosis and treatment via surgical fasciotomy.

Anatomically, the upper arm contains three compartments including the anterior (flexor), posterior (extensor), and the deltoid compartment. The deltoid compartment is innervated by the axillary nerve and surrounded by the deltoid fascia which splits into two parts. While the anterior compartment of the arm includes the brachialis, biceps brachii and coracobrachialis muscles. It is supplied by the brachialis artery and innervated by the musculocutaneous nerve. The median, ulnar, radial, medial antebrachial cutaneous, and lateral antebrachial cutaneous nerves also course distally in the anterior compartment [3,7].

The posterior compartment contains the profunda brachii artery and innervated by the radialis nerve. This compartment consists of two muscles, the triceps brachii and the anconeus muscle. The posterior antebrachial cutaneous nerve and the anconeus also crosses the posterior compartment [3,7,10,12].

There are multiple methods for decompression fasciotomy of the upper arm that has been described in the literature.

Fasciotomy of the anterior and posterior compartments of the arm uses one skin incision, in which a 15-cm skin incision is made over the medial intermuscular septum. Thereafter, the fascia over the anterior compartment is then opened midway between the anterior border of the biceps muscle, the posterior border of the triceps muscle, and the medial intermuscular septum for the length of the skin incision [1-3,7,10].

Two skin incisions approach to release the anterior and posterior compartments was also described in which a 15-cm skin incision starting medial to the bicipital sulcus is extended up the anteromedial arm to the acromion and through the fascia to decompress the anterior compartment. This is followed by another 15-cm skin incision starting at the tip of the olecranon and extended up the posterolateral arm and through the fascia to decompress the posterior compartment [3,7,12].

The forearm has three compartments: anterior (volar), posterior (dorsal) and the mobile WAD. The anterior compartment contains eight muscles which flex the wrist and fingers and is innervated mainly by the median nerve. The posterior compartment contains 12 muscles which are responsible for extension of the wrist, digits and supination of the forearm muscles, and is innervated by the radial nerve. Finally, the mobile WAD is a collective term for the lateral muscles brachioradialis, extensor carpi radialis brevis, and extensor carpi radialis longus. These three muscles act as flexors at the elbow joint allowing for elbow flexion and pronation.

There are three common methods for a forearm fasciotomy, most commonly the gentile S-shaped incision (Volar), volar-ulnar, and the separated incision.

The volar incision is made by an incision 1 cm proximal to the medial condyle that curves medially, reaching the midline at the junction of the middle and distal third of the forearm. Fasciotomy should release the fascia of the superficial layer, the deep layer that contains the pronator quadratus, and the deep flexor compartment. Using this incision technique, the dorsal compartment and the mobile extensor WAD can also be released without the need for a dorsal incision [3,7].

The forearm volar-ulnar approach starts with a transverse incision distal to the antecubital crease on the radial side of the forearm extended to the ulnar side of the forearm and then turned 90°. The longitudinal component of the incision is extended down the ulnar side of the forearm until it reaches the wrist, where it curves medially to the mid-aspect of the volar wrist. Finally, the incision is extended and curved into the thenar crease of the palm. By dividing the underlying fascia at the transverse origin of the incision distal to the antecubital crease, the muscles of the lateral (mobile wad) compartment are decompressed. The fascia underlying the longitudinal space between the FCU and FDS muscles (flexing the fingers will help differentiate these muscles) is separated with retractors, in which the ulnar nerve and artery are visualized lying on the deep flexor compartment [1-4,7].

The separated incision is a dorsal release approach by making a longitudinal incision dorsally beginning 3 to 4 cm distal to the lateral epicondyle and toward the Lister tubercle. It is possible to decompress all compartments of the dorsal compartment and mobile WAD in addition to part of the volar compartment with a volar radial incision [3,7].

The outcome after compartment syndrome depends on the duration of ischemia, severity of injury, associated injuries, and comorbidities. Myoneural necrosis can occur due to ischemia and due to low tissue pH as a result of lactic acidosis secondary to anaerobic metabolism and a release of K+. Rhabdomyolysis results in myoglobin release to the blood stream which can lead to acute tubular necrosis and acute renal failure, ultimately sepsis and death [1-4,9-12].

The most important factor to determine the degree of morbidity and mortality is the time from diagnosis to fasciotomy. Post-fasciotomy mortality rate is 11% to 15% and amputation rate of 11% to 21%. Generally, an irreversible muscle and nerve injuries occur after eight hours of ischemia. Any delay beyond 8 to 24 hours may result in Volkmann ischemic contracture, neurologic deficit, infection, amputation, or death [3,13].

The long-term sequelae of a fasciotomy were described by Fitzgerald et al. in which 60 patients were studied. Of those 60 patients 45 underwent lower extremity fasciotomies and 15 forearm fasciotomies. Finally, 25 patients underwent primary closure and 35 patients underwent split-thickness skin graft. This retrospective study concluded that 95% of those patients sustained a permanent altered sensation, 54% chronic limb pain, 40% dry skin, 30% discoloration of skin, 26% had contractures, 15% had edema and 13% had muscle herniation [9-12].

The main goal in the treatment of acute compartment syndrome is a decompressive fasciotomy of all affected muscle compartments, nerves, and vessels. Upper arm compartment syndrome is rare and must be suspected in patient with crushing injuries. Only in rare cases, both anterior and posterior compartments are involved and they both can be released through a single medial or lateral incision [3,7].

Post-fasciotomy, wounds are usually left open and a second operation is often performed within 48 to 72 h for further debridement and irrigation. Delayed primary wound closure can be done at the same time. Multiple other techniques and devices have been proposed to increase the rate of skin closure and reduce the need for skin grafting such as Shoelace Technique, Mechanical Devices (Suture Tension Adjustment Reel and the Dynamic Wound Closure Device), vacuum-assisted closure, and skin grafting [12,14-16].

## Conclusions

An upper arm compartment syndrome is rare but should be recognized promptly as any delay in diagnosis and treatment can result in high morbidity and mortality. There are multiple causes of an upper arm compartment syndrome, most commonly described in the literature are crushing injuries, condylar and supracondylar fractures, tourniquet at the upper arm, and after venipuncture. Any patient with severe swelling, pain out of proportion, or any neurovascular deficits should be evaluated for a compartment syndrome. When in doubt, there are various methods to measure a compartment pressure. Although it is rare to have anterior and posterior compartment syndrome at the same time both compartments must be assessed and released. There are multiple surgical methods to release an upper arm and forearm compartments. Surgeons should have low threshold for performing upper arm and forearm fasciotomy when suspecting a compartment syndrome, especially in patients with crushing injuries of the arm and forearm with associated injuries such as bone fractures.
